# Electromagnetic Modeling and Structure Optimization of a Spherical Force Sensing System

**DOI:** 10.3390/s19030552

**Published:** 2019-01-29

**Authors:** Liang Yan, Yinghuang Liu, Zongxia Jiao

**Affiliations:** 1 School of Automation Science and Electrical Engineering, Beihang University, Beijing 100191, China; tonnnyala@126.com (Y.L.); zxjiao@buaa.edu.cn (Z.J.); 2 Shenzhen Institute of Beihang, Shenzhen 518057, China

**Keywords:** force sensing system, adaptive PSO algorithm, electromagnetic modeling

## Abstract

Force sensing system (FSS) is widely used to simulate the control force of aircrafts for pilots. Conventional FSS employs multiple single-axis motors and complex transmission mechanisms to achieve multiple degree-of-freedom (DOF) force output of joystick, which may cause mismatched inertia and affect the output performance of FSS significantly. Therefore, one novel FSS with multiple DOF direct-drive spherical actuator is proposed in this paper to reduce the simulator’s extra inertia. To analyze its output performance systematically, a hybrid modeling method is proposed to formulate both Ampere torque and cogging torque mathematically. Equivalent current method along with Ampere force law is used to obtain the Ampere torque due to irregular structure of magnet and coil poles. The cogging torque is then obtained from airgap flux density via Maxwell stress method. From the derived analytical model, an adaptive particle swarm optimization (PSO) algorithm based on expectation (the average value of minimum errors) is proposed for multiple-parameter structure optimization. It can avoid local optimization effectively. The study shows that the optimized value greatly helps to improve the torque generation. Then, one research prototype and one testbed is developed. The comparison between experimental result and analytical model shows that the two sets of data fit with each other well. Therefore, the analytical model could be employed for motion control of the system at the next stage.

## 1. Introduction

Force sensing system (FFS) is an aircraft utility device to provide pilots with the sense of control force feedback from the rudder load [1,2,3]. It plays an extremely important role in generating the most intuitive information of aircraft status [4,5,6]. In flight simulators, the performance of FSS directly affects the training result of pilots and the performance evaluation of aircrafts. FSS is generally categorized into digital hydraulic and digital electric types [7,8]. Compared with the digital hydraulic system, the digital electric system can effectively reduce energy loss and environmental pollution. It is the major development trend of FSS in the future [9,10,11]. The digital electric FSS designed by Prendergast et al. is composed of a linear motor, digital computer, and position loop force sensor, etc. However, the employment of the linear motor driver increases the system volume and mass [12]. Wang et al. developed an electric FSS in which the motor adjusts the system stiffness according to the force model to control the joystick force sense [13,14]. Due to the complexity of the system, the inertia force is large. However, damping force and inertia force are not studied.

The inertia-redundant force greatly affects the performance of FSS [15,16]. Traditional FSSs require multiple single-axis motors, connecting rods, gears, worms and other components [17,18]. The complex structure yields large inertia-redundant force, and thus compromises the output performance of servo system unavoidably.

Therefore, this paper proposes a novel FSS driven by spherical actuator with two-dimensional (2D) camber Halbach array to reduce the inertia-redundant force [19,20,21]. Spherical actuator is an electric device capable of achieving multiple DOF rotary motions in a single joint [22,23,24]. Compared with conventional FSS, it has compact structure [25,26], lower moment inertia, and fast response [27,28]. A hybrid approach is proposed to formulate the torque output of the spherical actuator analytically. The equivalent current method along with Ampere force law is used to obtain the Ampere torque due to irregular structures of magnet and coil poles. The cogging torque is then obtained from airgap flux density between rotor and stator via Maxwell stress method. Following that, based on the derived analytical model, an adaptive particle swarm optimization (PSO) algorithm [29,30] based on expectation is proposed for multiple-parameter structure optimization. For traditional PSO algorithms, it is difficult to confirm particle number before optimization, which may lead to local optimum value instead of global value [31,32]. The proposed adaptive PSO algorithm can modify particle quantity based on iterating effect, and achieve global optimum value. The simulation results show that it can solve the problem of particle number initialization, and the optimized values can improve the system output performance greatly. Based on the optimum parameter values, one research prototype and one testbed are developed. Experiments are conducted on the torque generation of the FSS. It shows that the analytical model fits with the experimental results well. Therefore, the validated analytical torque solution could be used for future studies on motion-control implementations. The comparison with traditional spherical actuator shows that the novel design with 2D camber Halbach array greatly improves the output torque. It also provides torque ripple that allows pilots to perceive the variation of rudder load. The comparison further verifies that the novel FSS can reduce the inertia-redundant force effectively.

The rest of this paper is organized as follows. Section 2 presents the design concept and operating principle of the proposed spherical actuator. Section 3 formulates the output torque of 2D longitudinal camber Halbach array analytically. Section 4 optimize the electromagnetic structure parameters based on the adaptive PSO algorithm. Section 5 evaluates torque increase and inertia decrease of the FSS. Section 6 presents the development of research prototype and testbed, and then experimental studies are carried out to validate the analytical solution. Section 7 concludes the research work.

## 2. Schematic Structure and Operating Principle

**Figure 1:**
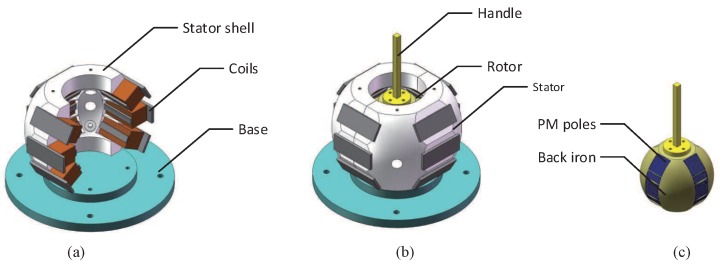
Structure of FSS with spherical actuator. (**a**) stator; (**b**) overview construction; (**c**) rotor.

As shown in Figure 1, the schematic structure of the spherical actuator with 2D camber Halbach array and iron stator for FSS of flight simulators is presented. It is mainly composed of one spherical shell-like stator and one rotor housed inside. Four sets of permanent magnet (PM) poles are mounted and evenly distributed along the circumferential direction of the rotor. Each set includes five poles magnetized in radial and tangential directions constituting 2D camber Halbach array on a spherical surface. Similarly, four sets of coils are mounted on the stator along the circumference of the stator, each consisting of three coils in the longitudinal direction.

**Figure 2:**
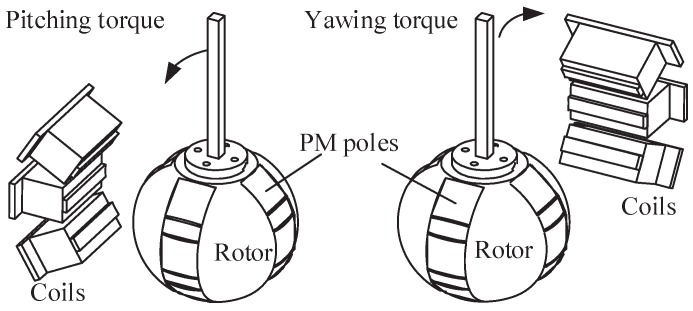
Operating principle of spherical actuator.

As shown in Figure 2, the operating principle of the proposed spherical actuator is illustrated. The interaction between magnetic field of rotor poles and current input of stator coils generates attraction or repulsion forces, and thus produces inclination torque on the rotor. Ferromagnetic material is employed for the stator and rotor design to reduce the magnetic energy loss and thus improve the output performance. It is especially useful for the implementation of joysticks in aircrafts.

## 3. Torque Modeling of Force Sensing System

### 3.1. Magnetic Field Model with Full Sets of Magnets

**Figure 3:**
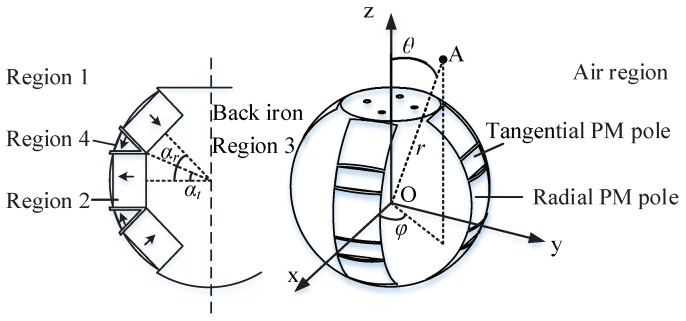
Distribution of PM poles array.

As shown in Figure 3, to define a spherical coordinate system [22], we choose two orthogonal directions, the zenith and an origin point at the rotor center. This choice determines a reference plane that contains the origin and is perpendicular to the zenith. The spherical coordinates of a point *A* are then defined as follows: the radial distance *r* is the distance from the origin *O* to *A*; the inclination (or polar angle) θ is the angle between the *z* axis and the line segment OA; the azimuth (or azimuthal angle) φ is the signed angle measured from the *x* axis to the orthogonal projection of the line segment OA on the reference plane.

As shown in Figure 2 and Figure 3, to formulate the magnetic field distribution, the actuator space under study is divided into four regions: (1) outer air layer; (2) radial PM poles; (3) back iron of rotor; (4) tangential PM poles. The full sets of magnets are considered simultaneously to formulate the magnetic field distribution around the rotor surface.

We assume that the ferromagnetic material works in the linear portion without hysteresis, and the permeability of ferromagnetic material is much greater than that of air gap [33]. Considering the boundary condition between radial and tangential poles, spherical harmonics function can represent the magnetic flux density of magnetic poles. Therefore, the three components of the magnetic flux density can be obtained as

(1) $$\begin{array}{c} B_{1,r}=\frac{15\mu_{0}}{8\pi }d_{\xi ,4}r^{-6}\sqrt{\frac{35}{2}}\sin^{4}\varphi \left[M_{r}a_{r,m}c_{r,m}\cos4\theta \right.\left.+M_{t}a_{t,m}c_{t,m}\cos4\left(\theta -\alpha_{1}\right)\right],\\ B_{1,\theta }=\frac{3\mu_{0}}{2\pi }\sqrt{\frac{35}{2}}d_{\xi ,4}r^{-6}\sin^{3}\varphi \left[M_{r}a_{r,m}c_{r,m}\sin4\theta \right.\left.+M_{t}a_{t,m}c_{t,m}\sin4\left(\theta -\alpha_{1}\right)\right],\\ B_{1,\varphi }=\frac{3\mu_{0}}{2\pi }\sqrt{\frac{35}{2}}d_{\xi ,4}r^{-6}\sin^{3}\varphi \cos\varphi \left[M_{r}a_{r,m}c_{r,m}\cos4\theta \right.\left.+M_{t}a_{t,m}c_{t,m}\cos4\left(\theta -\alpha_{1}\right)\right]. \end{array}$$

where $B_{1,r}$, $B_{1,\theta }$, $B_{1,\varphi }$ are three components of magnetic flux density produced by the PM pole; $M_{r}$ and $M_{t}$ are residual magnetization of radial and tangential PMs; $\mu_{0}$ is permeability of free space; $\alpha_{1}$ is angle between radial and tangential PM, $d_{\xi ,4}$, $a_{r,m}$, $c_{r,m}$, $a_{t,m}$, and $c_{t,m}$ are coefficients obtained based on the spherical harmonic equation and boundary conditions.

**Figure 4:**
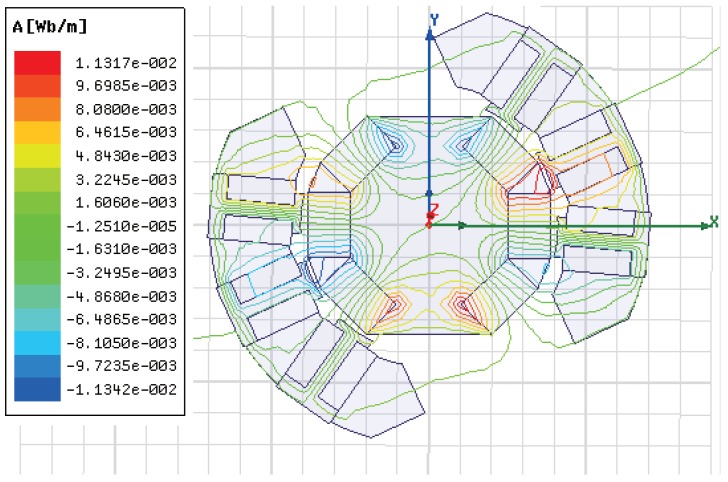
The cross-sectional drawing of the magnetic circuit.

The cross-sectional drawing of the magnetic circuit is shown in Figure 4. It can be found that the magnetic flux generated by PMs forms a close loop through the magnets, the air gap, the coils, the stator shell, and the rotor core. Only small amount of magnetic leakage is not connected by back iron. The magnetic flux energized by coils follows similar distribution between the rotor and the stator. The employment of the rotor iron core and the stator iron shell helps to reduce magnetic leakage and improve the system output performance.

To obtain the output torque generated by one coil, we assume the coil consists of numerous differential segments. It can be verified that only the radial component $B_{1,r}$ can produce magnetic torque. $B_{1,\theta }$ and $B_{1,\varphi }$ do not produce magnetic torque on the rotor, because the Ampere force generated by them passes through the rotor center. The total torque by one coil can be obtained by integrating the differential torque in the coil volume. The similar approach is employed by other researchers [34] In addition, based on the principle of Maxwell stress equation, cogging torque is generated by $B_{1,\theta }$ and $B_{1,r}$. Therefore, the subsequent torque study will focus on $B_{1,r}$ and $B_{1,\theta }$.

### 3.2. Torque Generated by Single Coil

To facilitate the calculation of Ampere force, the stator windings are equivalent to magnets and PMs are equivalent to coils. The coil assumes a conical-shaped object embedded in the stator shell to facilitate the formulation of the actuator torque [35]. The magnetic flux density generated by the stator winding is

(2) $$B_{c}=\frac{1}{S_{3}}\cdot \frac{NI_{c}}{\frac{l_{1}}{\mu_{1}S_{1}}+\frac{l_{2}}{\mu_{2}S_{2}}},$$

where $S_{1}$, $S_{2}$ and $S_{3}$ are respectively the magnetic flux area of the iron core, coil and winging, $\mu_{1}$ and $\mu_{2}$ are respectively the permeability of iron core and coil, $l_{1}$ and $l_{2}$ are respectively the length of iron core and coil, and *N* is the number of winding turns. $I_{c}$ is the coil current shown as follows:

(3) $$I_{c}=i\cdot sign\left(-\theta \right),$$

where *i* is magnitude of current input, θ is angular displacement of actuator. In PM pole,

(4) $$\begin{array}{c} J_{m}=\nabla \times B_{m}, \end{array}$$

where $J_{m}$ is equivalent surface current density of PM pole, and $B_{m}$ is flux density of PM pole. Let $B_{m}=\left|B_{m}\right|$, $J_{m}=\left|J_{m}\right|$, thus,

(5) $$\begin{array}{c} dI_{m}=J_{m}ds, \end{array}$$

where $dI_{m}$ is differential equivalent surface current of PM pole, and ds is differential area of PM pole. Hence, we have

(6) $$\begin{array}{c} F_{\theta_{i}}=J_{m}S_{\theta }l_{m}\frac{NI_{c}}{S_{3}(\frac{l_{1}}{\mu_{1}S_{1}}+\frac{l_{2}}{\mu_{2}S_{2}})}, \end{array}$$

where $F_{\theta_{i}}$ is Ampere force of *i*-th PM pole in θ angular displacement, $S_{\theta }$ is equivalent cross-sectional area in θ angular displacement, $l_{m}$ is equivalent segment of PM pole [36,37]. Thus, the torque generated by a single coil can be obtained as

(7) $$T_{\theta_{i}}=J_{m}S_{\theta }l_{m}R\frac{NI_{c}}{S_{3}\left(\frac{l_{1}}{\mu_{1}S_{1}}+\frac{l_{2}}{\mu_{2}S_{2}}\right)},$$

(8) $$T_{\varphi_{i}}=J_{m}S_{\varphi }l_{m}R\frac{NI_{c}}{S_{3}\left(\frac{l_{1}}{\mu_{1}S_{1}}+\frac{l_{2}}{\mu_{2}S_{2}}\right)},$$

where $T_{\theta_{i}}$ and $T_{\varphi_{i}}$ are components of torque, $S_{\varphi }$ is equivalent cross-sectional area in φ angular displacement, and *R* is rotor radius. Since the radial force does not produce torque, we have where torque component $T_{r_{i}}$

(9) $$T_{r_{i}}=0.$$

Thus, the total electromagnetic torque by a single coil is

(10) $$T_{i}=\left[\begin{array}{c} T_{r_{i}}\\ T_{\theta_{i}}\\ T_{\varphi_{i}} \end{array}\right]=\left[\begin{array}{c} 0\\ J_{m}S_{\varphi }l_{m}R\frac{NI_{c}}{S_{3}\left(\frac{l_{1}}{\mu_{1}S_{1}}+\frac{l_{2}}{\mu_{2}S_{2}}\right)}\\ J_{m}S_{\theta }l_{m}R\frac{NI_{c}}{S_{3}\left(\frac{l_{1}}{\mu_{1}S_{1}}+\frac{l_{2}}{\mu_{2}S_{2}}\right)} \end{array}\right].$$

### 3.3. Cogging Torque of Single Coil

According to Maxwell’s stress equation, we can obtain two formulas of magnetic force between magnets as

(11) $$F_{n}=\frac{1}{2\mu_{0}}\int \int \left(B_{n}^{2}-B_{t}^{2}\right)dS,$$

(12) $$F_{t}=\frac{1}{\mu_{0}}\int \int B_{n}B_{t}dS,$$

where $F_{n}$ and $F_{t}$ are respectively the normal and tangential components of the magnetic field force, dS is differential area of coil cross section and $B_{n}$ and $B_{t}$ are respectively the normal and tangential components of the magnetic flux density. In this study, the magnetic field between the iron core and the PM generates the magnetic cogging force. Since the radial magnetic field force does not produce torque, we only consider the tangential force. We can simplify the Maxwell stress formulation as follows:

(13) $$F_{t}=\frac{1}{\mu_{0}}\int \int B_{n}B_{t}dS=\frac{1}{\mu_{0}}\int \int B_{m}cos\gamma B_{m}sin\gamma dS,$$

where $B_{m}$ is the magnetic flux density of PM pole, γ is the angle between the PM pole and *z*-axis. Thus,

(14) $$\begin{array}{c} F_{f_{i}}=\frac{1}{\mu_{0}}\int \int B_{m}cos\gamma B_{m}sin\gamma dS=\frac{1}{\mu_{0}}B_{r_{i}}B_{\theta_{i}}S_{3}, \end{array}$$

where $F_{f_{i}}$ is magnetic cogging force of *i*-th iron core, $B_{r_{i}}$ and $B_{\theta_{i}}$ are respectively the normal and tangential components of the magnetic flux density of *i*-th iron core, and $S_{3}$ is the magnetic flux area of winging. Thus, the torque generated by a single winding can be obtained as

(15) $$T_{f_{i,\theta }}=\frac{1}{\mu_{0}}RB_{r_{i}}B_{\theta_{i}}S_{3},$$

(16) $$T_{f_{i,\varphi }}=\frac{1}{\mu_{0}}RB_{r_{i}}B_{\varphi_{i}}S_{3},$$

where $T_{f_{i,\theta }}$ and $T_{f_{i,\varphi }}$ are components of cogging torque, $B_{r_{i}}$, $B_{\theta_{i}}$ and $B_{\varphi_{i}}$ are three components of PM magnetic flux density, and *R* is rotor radius. Since the radial magnetic field force does not produce torque, we obtain radial torque as

(17) $$T_{f_{i,r}}=0.$$

Thus, the total cogging torque by a single coil $T_{f_{i}}$ is

(18) $$T_{f_{i}}=\left[\begin{array}{c} T_{f_{i,r}}\\ T_{f_{i,\theta }}\\ T_{f_{i,\varphi }} \end{array}\right]=\left[\begin{array}{c} 0\\ \frac{1}{\mu_{0}}RS_{3}B_{r_{i}}B_{\theta_{i}}\\ \frac{1}{\mu_{0}}RS_{3}B_{r_{i}}B_{\varphi_{i}} \end{array}\right].$$

### 3.4. Torque Generated by Full Set of Coils

By summing whole coils’ torque $T_{\theta_{i}}$ based on the Ampere formula, we obtain the torque in θ direction is

(19) $$T_{\theta }=2J_{m}l_{m}RB_{c}\left(S_{\theta_{1}}+S_{\theta_{2}}+S_{\theta_{3}}\right),$$

where $S_{\theta_{1}}$, $S_{\theta_{2}}$ and $S_{\theta_{3}}$ are equivalent cross-sectional area $S_{\theta }$ of *i*-th PM. Thus, the *x*-axis component of the electromagnetic torque of the entire actuator is

(20) $$\begin{array}{c} T_{x}=2J_{m}l_{m}RB_{c}\left(S_{\theta_{1}}+S_{\theta_{2}}+S_{\theta_{3}}\right){\left[1\;\;0\;\;0\right]}^{T}, \end{array}$$

where

(21) $$\left[\begin{array}{c} \theta_{1}\\ \theta_{2}\\ \theta_{3} \end{array}\right]=\left[\begin{array}{c} \theta +\frac{1}{3}\pi \\ \theta +\frac{1}{2}\pi \\ \theta +\frac{2}{3}\pi \end{array}\right].$$

Therefore, the output torque of the actuator is

(22) $$\begin{array}{c} T_{x}+T_{x_{f}}=2J_{m}l_{m}RB_{c}\sum_{i=1}^{3}S_{\theta_{i}}{\left[\begin{array}{ccc} 1 & 0 & 0 \end{array}\right]}^{T}+\frac{2}{\mu_{0}}RS_{3}B_{f}{\left[\begin{array}{ccc} B_{\theta_{1}} & B_{\theta_{2}} & B_{\theta_{3}} \end{array}\right]}^{T}{\left[\begin{array}{ccc} 1 & 0 & 0 \end{array}\right]}^{T}, \end{array}$$

where $T_{x_{f}}$ is the *x*-axis component of total cogging torque, $B_{f}=\left[\begin{array}{ccc} B_{r_{1}} & B_{r_{2}} & B_{r_{3}} \end{array}\right]$, $B_{r_{1}}$, $B_{r_{2}}$ and $B_{r_{3}}$ are components of PM magnetic flux density in *r* direction, and $B_{\theta_{1}}$, $B_{\theta_{2}}$ and $B_{\theta_{3}}$ are components of PM magnetic flux density in θ direction. Equation (22) represents the torque component in single direction. The overall electromagnetic torque is

(23) $$\begin{array}{c} T_{e}=\left[\begin{array}{c} 2J_{m}l_{m}RB_{c}\sum_{i=1}^{3}S_{\theta_{i}}\\ 2J_{m}l_{m}RB_{c}\sum_{i=4}^{6}S_{\varphi_{i}}\\ 0 \end{array}\right]. \end{array}$$

Considering the cogging torque of the actuator, the actual output torque of the 2-DOF actuator is

(24) $$T_{o}=\left[\begin{array}{c} 2J_{m}l_{m}RB_{c}\sum_{i=1}^{3}S_{\theta_{i}}+\frac{2}{\mu_{0}}RS_{3}B_{f}{\left[B_{\theta_{1}}B_{\theta_{2}}B_{\theta_{3}}\right]}^{T}\\ 2J_{m}l_{m}RB_{c}\sum_{i=4}^{6}S_{\varphi_{i}}+\frac{2}{\mu_{0}}RS_{3}B_{f}{\left[B_{\theta_{4}}B_{\theta_{5}}B_{\theta_{6}}\right]}^{T}\\ 0 \end{array}\right].$$

## 4. Structure Optimization Based on Adaptive PSO Algorithm

### 4.1. Adaptive PSO Algorithm with Expectation and Deviation

#### 4.1.1. Traditional PSO Algorithm

Traditional PSO can be expressed as follows: assume a particle swarm composed of *m* particles in a dimensional search space; the position of particle *i* is defined as $X\left(i\right)=\left(x_{i1},x_{i2},x_{iD}\right),i=1,\;2,\;\cdots m$, where *D* is dimension; the individual optimal position is $P_{i}$, its fitness is $F_{i}$, and its speed is $V_{i}$. The global optimal position is $P_{g}$. In this paper, optimization parameters are defined as $D=3,x_{i1}=S_{1},x_{i2}=S_{2}$ and $x_{i3}=S_{3}$. Then, the speed $v_{i}^{n+1}$ and position $x_{i}^{n+1}$ of the *i*-th particle in generation n+1 can be calculated iteratively according to the following equations:

(25) $$\begin{array}{c} v_{i}^{n+1}=wv_{i}^{n}+c_{1}r_{1}\left(P_{i}^{n}-x_{i}^{n}\right)+c_{2}r_{2}\left(P_{g}^{n}-x_{i}^{n}\right), \end{array}$$

(26) $$\begin{array}{c} x_{i}^{n+1}=x_{i}^{n}+v_{i}^{n+1}, \end{array}$$

where *w* is inertial weight, $c_{1}$, $c_{2}$ are acceleration coefficients that usually have the same value, and $r_{1},r_{2}$ are two random values in the range of [0, 1]. In the optimization, the speed of the particle is usually limited to a range with $v_{max}$ as its critical value, and the position of the particle is also limited within a certain range. In addition, during the iteration, $P_{i}$ and $P_{g}$ are constantly updated so that the optimal solution of $P_{g}$ can be obtained.

#### 4.1.2. Adaptive PSO Algorithm with Anti-Local Optimization

**Figure 5:**
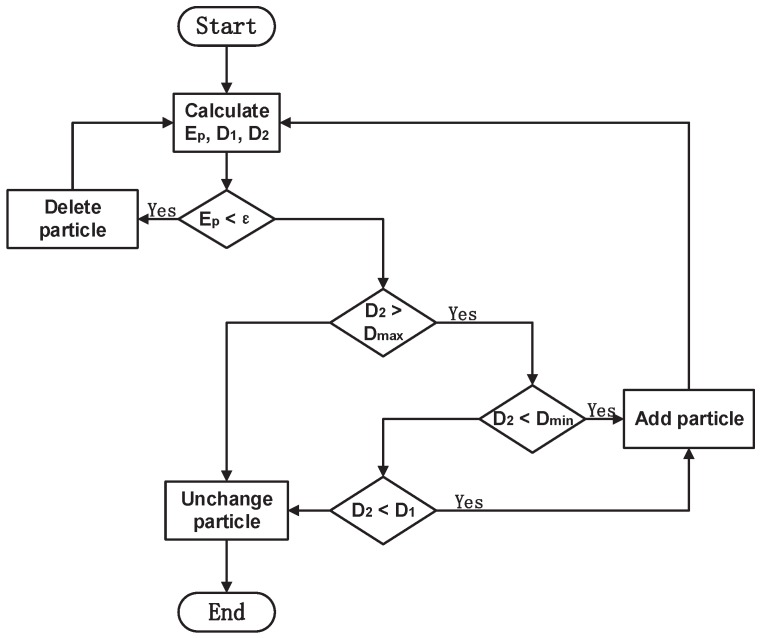
Adaptive PSO algorithm structure.

In traditional PSO algorithm, the particle quantity is difficult to be determined. The calculation result may not meet the accuracy requirement due to improper selection of particle quantity. Therefore, a novel adaptive PSO algorithm with anti-local optimization is proposed. An external nested loop algorithm is embedded to increase or decrease particles quantity in PSO iteration. Then, the PSO algorithm conducts iteration in double learning loops, gradually optimizing the number of particles, at the same time modifying the final error to meet the optimization requirement. The structure of the proposed adaptive algorithm is shown in Figure 5.

The modification rule of particle quantity in the adaptive PSO algorithm is as follows. In the first place, the initial particle quantity in the second layer of PSO algorithm is set by considering the actual situation, in this case 5. Then, the PSO algorithm iterates to modify the number of particles (increase or decrease), until it reaches iteration requirement. The PSO algorithm expectation definition is given as

(27) $$E_{p}=\frac{\sum_{p=1}^{p}\sum_{k=2}^{N_{L}}\left(Y_{k}^{p}\left(L\right)-Y_{k-1}^{p}\left(L\right)\right)}{P},$$

where *P* is the total number of particles, *L* is the particle layer of PSO algorithm, $N_{L}$ is the generation number of PSO algorithm, *Y* is the actual output. The expectation is average value of minimum errors and is used to evaluate whether the PSO algorithm achieves the optimization goal. When the PSO iteration number meets the requirement, the attenuation rate *D* is defined as

(28) $$D=\frac{E_{p}\left(T-1\right)-E_{p}\left(T\right)}{E_{p}\left(T-1\right)}.$$

In this study, the cost function is

(29) $$l_{\min}=Min\left|l_{len}-Max\left(T_{o}\right)\right|,$$

where $l_{\min}$ is minimum distance, $l_{len}$ is initial constant. The constraint of PSO is

(30) $$l_{\min}\left(T\right)-l_{\min}\left(T+1\right)<\varepsilon ,$$

where ε is error threshold. Define $D_{1}$, $D_{2}$ as the declining rates before and after *T*-th iteration, $D_{s}$, $D_{f}$ as the predetermined attenuation rates, and $D_{\max}>D_{\min}>0$, ε>0. There are several addition and deletion rules:

- Initialize the parameters of PSO algorithm randomly. When the number of iterations reaches *T*, calculate $E_{p},D_{1},D_{2}$;
- If $E_{p}<\varepsilon$, i.e., PSO algorithm is convergence, delete 10 particles;
- If $D_{2}>D_{\max}$, i.e., PSO algorithm performance is perfect, maintain the PSO structure;
- If $D_{2}<D_{\min}$, i.e., PSO performance is poor, increase 10 particles;
- If $D_{\min}<D_{2}<D_{\max}$ and $D_{2}<D_{1}$, increase 10 particles; otherwise, maintain the PSO structure.

### 4.2. Structural Parameter Optimization

**Figure 6:**
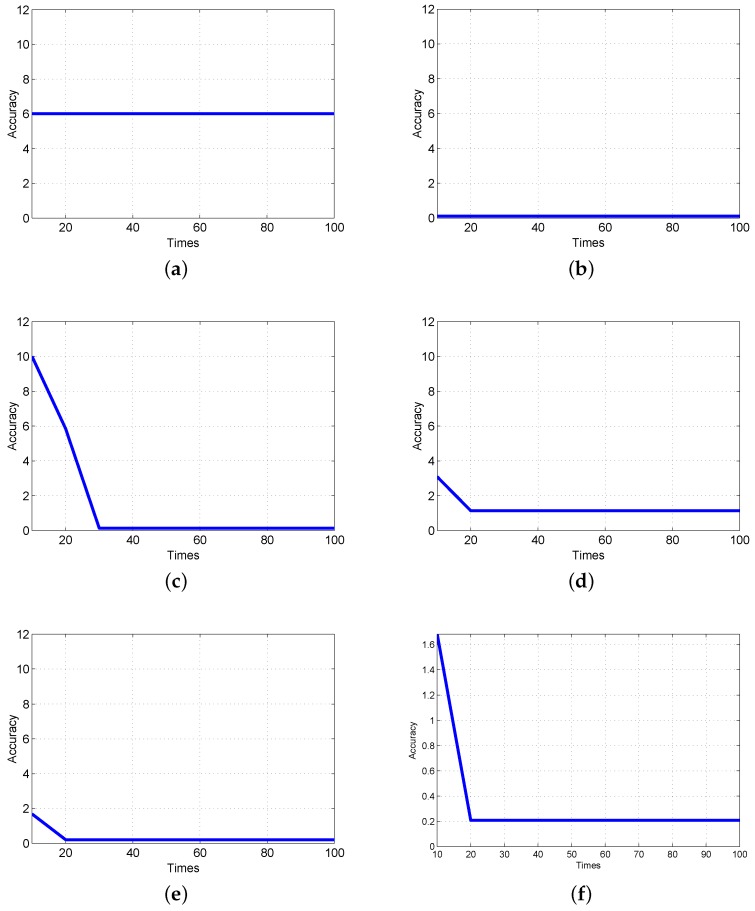
Optimization accuracy curve based on adaptive PSO algorithm. (**a**) Optimization accuracy with 5 particles; (**b**) Optimization accuracy with 15 particles; (**c**) Optimization accuracy with 25 particles; (**d**) Optimization accuracy with 25 particles; (**e**) Optimization accuracy with 35 particles; (**f**) Optimization accuracy with 35 particles.

The iteration accuracy curves of PSO from 5 to 35 particles are shown in Figure 6. We find that the accuracy of Figure 6b,c satisfies the requirements, but the algorithm continues to iterate. It indicates that there is a local minimum value. In addition, although PSO particle number of Figure 6c is consistent with that of Figure 6d, parameters in algorithm are not the same. Therefore, the results of two iteration accuracy curve are similar but slightly different. At last, PSO with 35 particles meet the optimization accuracy demand.

**Figure 7:**
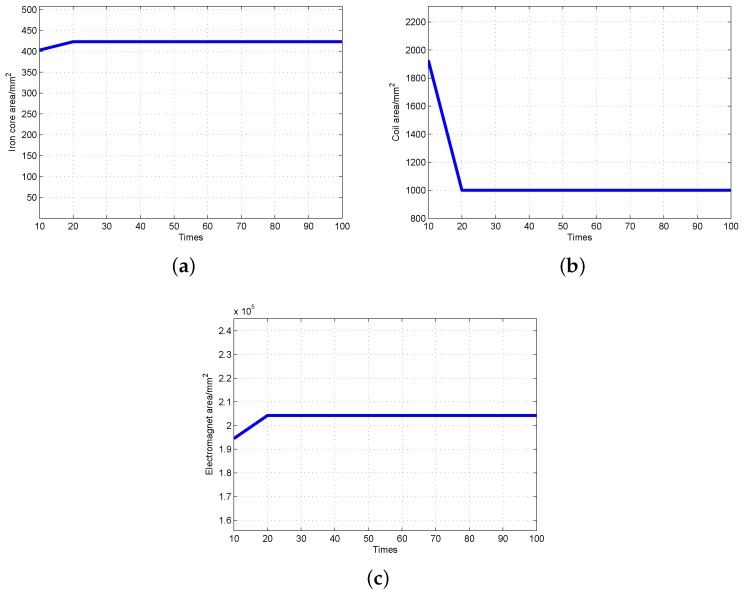
Optimization of electromagnetic structure parameters. (**a**) Optimization of iron core area; (**b**) Optimization of coil area; (**c**) Optimization of electromagnet area.

The calculation results are shown in Table 1. It includes PSO algorithm expectation $E_{p}$, decline rate $D_{1}$ and $D_{2}$. Let the largest decline rate and minimum decline rate be 0.5 and 0.4, respectively. As shown in Table 1, the PSO iteration error rate is less than 0.4. The expectation generated by PSO iteration meets requirements until particles increase from 5 to 35. At the same time, the adaptive PSO stops the iteration. Therefore, the adaptive PSO can determine the particles quantity and avoid local optimization, which deduces the iterations of PSO. The optimization result of major electromagnetic structure parameters is shown in Figure 7.

**Figure 8:**
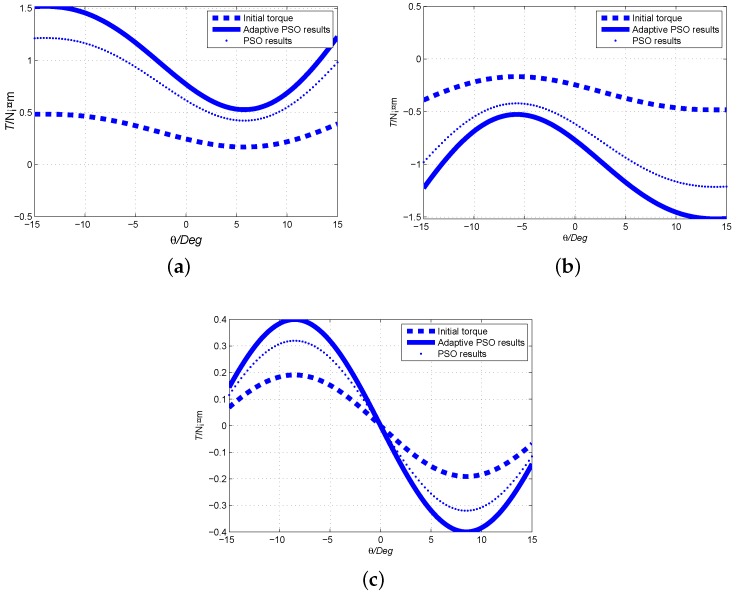
Optimization comparison of output torque. (**a**) Optimization comparison of output torque $T_{o+}$ when current direction is positive; (**b**) Optimization comparison of output torque $T_{o-}$ when current direction is negative; (**c**) Optimization comparison of output torque $T_{f}$.

The electromagnetic parameters of the initial, PSO and Adaptive PSO designs are calculated, and the torque outputs are compared. The comparisons of output torque $T_{o+}$(positive current), $T_{o-}$(negative current) and cogging torque $T_{f}$ are presented in Figure 8. It proves that the electromagnetic torque and cogging torque optimized by Adaptive PSO are better greater than those of initial design. Furthermore, the nonlinearity is found in the torque curve. Because the coil core is made of ferromagnetic material, the torque fluctuation is larger than that of air-core coil. In the case of no current supply, the torque is equal to zero at $\theta =0^{{}^{\circ}}$ because the Ampere torque is zero and the direction of the cogging torque is opposite at two sides of rotor equator. When the coils are supplied with power, the superposition of Ampere torque and cogging torque at two sides of rotor equator is no longer equal. Therefore, the resulting torque is not symmetrical around the zero position.

## 5. Performance Evaluation of the Proposed Design

### 5.1. Comparison of Torque Output between 2D Longitudinal Camber Halbach Array and Traditional PM Array

**Figure 9:**
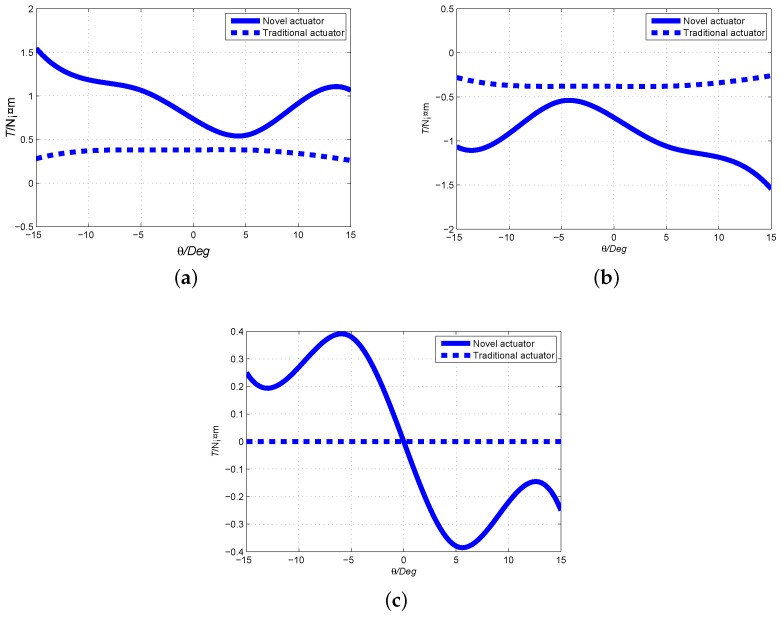
Comparison of output torque between 2D camber Halbach array and traditional PM array. (**a**) Output torque $T_{o+}$ when current direction is positive; (**b**) Output torque $T_{o-}$ when current direction is negative; (**c**) Output cogging torque $T_{f}$.

The torque output of 2D longitudinal camber Halbach PM array with ferromagnetic structure is compared with that of traditional alternative radial magnetization PM array with air-core coil. The major numerical model parameters is shown in Table 2. The comparison results of $T_{o+}$(positive current), $T_{o-}$(negative current) and cogging torque $T_{f}$ with respect to the longitudinal angle at $\varphi =90^{{}^{\circ}}$ are presented in Figure 9. It is found that the torque output of the proposed design is much larger than that of traditional design. In the torque comparison, we adopt the same current, magnetic pole volume, rotor volume, and airgap size. Because the novel spherical actuator’s coil core is made of ferromagnetic materials, and the PM array adopts 2D camber Halbach array, the torque output is greatly increased.

Generally, nonlinearity is not preferred for the design of electric actuators. However, the spherical actuator proposed in this paper is applied to the FSS of the flight simulator. The variation of torque with respect to the rotor orientation helps the pilot to feel the external loading from rudders. Therefore, the nonlinearity of output torque is necessary.

### 5.2. Inertia Moment Comparison between New FSS and Traditional FSS

**Figure 10:**
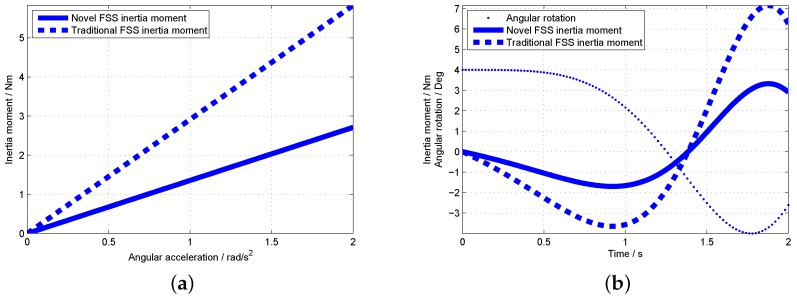
Inertia moment comparison of novel and traditional FSS. (**a**) Inertia moment under different angular acceleration; (**b**) Inertia moment changed with motion.

The inertia moment comparison between new FSS and traditional FSS is shown in Figure 10. Figure 10a shows that with the increase of angular acceleration, the inertia moment of the novel FSS is smaller than that of the traditional FSS. Figure 10b shows that the inertia moment of the new FSS is smaller than that of the traditional FSS with the increase of the motion frequency of the joystick. Therefore, the novel FSS with compact structure greatly reduce the inertia-redundant force caused by the complex transmission mechanism of the traditional FSS.

## 6. Experimental Investigation of Force Sensing System

### 6.1. Research Prototype and Testbed

**Figure 11:**
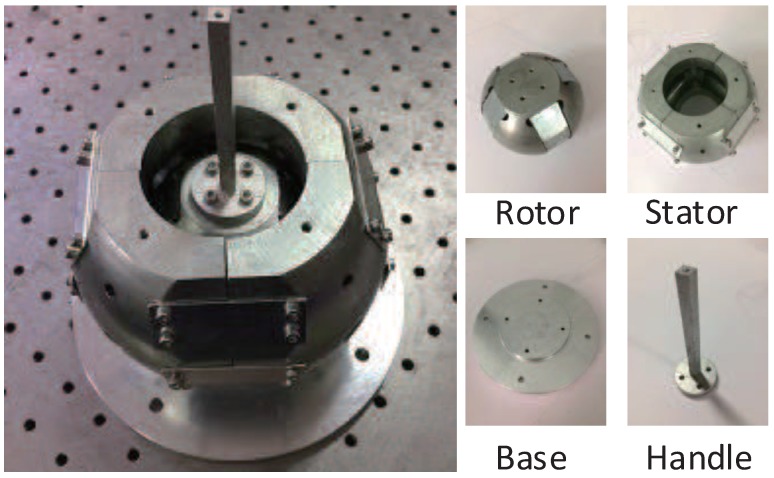
Overview of the developed research prototype.

One research prototype is developed as shown in Figure 11. The ball-like rotor is mounted with magnet poles on the surface, and the spherical-shell-like stator is mounted with coils. The rotor is housed inside the stator, and the interaction between magnet poles and the current input in the coils generates torque to move the rotor.

**Figure 12:**
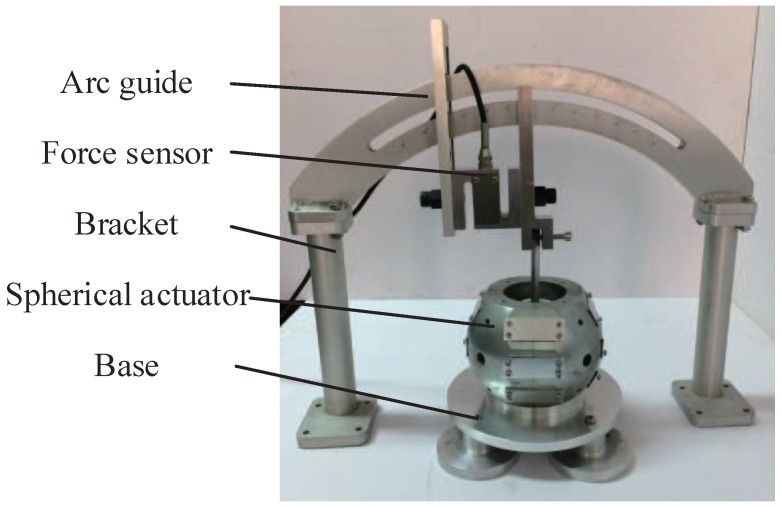
Experimental testbed for the measurement of output torque.

The experimental testbed is shown in Figure 12. The handle of the spherical actuator is mounted with force sensor which in turn is fixed on the arc guide. The handle can change its orientation along with the force sensor, and the torque output can be measured accordingly.

### 6.2. Comparison of Experimental Results and Analytical Model

**Figure 13:**
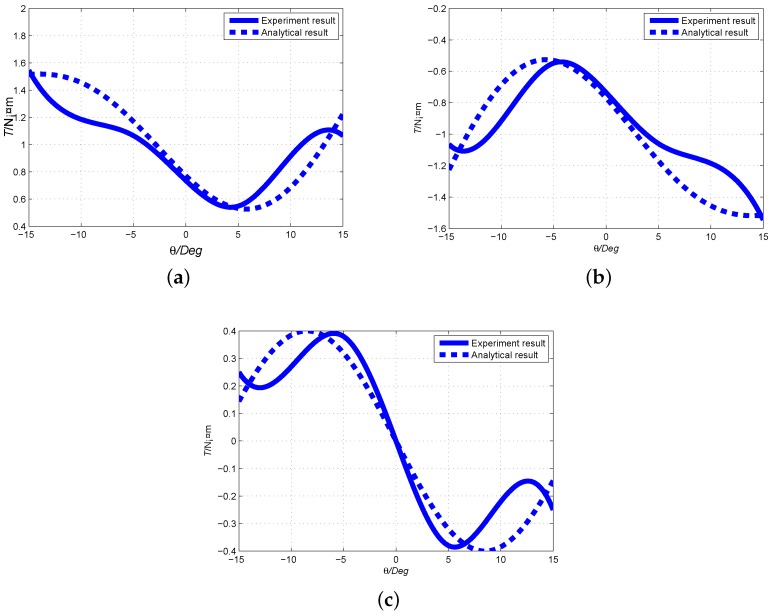
Comparison of output torque between experiment result and analytical result. (**a**) Output torque $T_{o+}$ when current direction is positive; (**b**) Output torque $T_{o-}$ when current direction is negative; (**c**) Output cogging torque $T_{f}$.

To further observe the variation of torque output and validate the analytical torque model, the experimental results are compared with the analytical model. The same structural parameters are employed for experimental and analytical computations. The comparisons of output torque $T_{o+}$(positive current), $T_{o-}$(negative current) and cogging torque $T_{f}$ are presented in Figure 13.

Figure 13a shows the comparison of analytical model and experimental result of output torque $T_{o+}$ when current direction is positive. In the longitudinal or θ direction, the variation of output torque creates one peak and one trough values, which is consistent with the magnet arrangement on the rotor surface. The peak values appear at $\theta =-15^{{}^{\circ}}$ and trough at $\theta =-5^{{}^{\circ}}$. It is also found that the analytical model fits with the experimental result of the output torque well. Similarly, Figure 13b presents the variation comparison of analytical model and experimental result of output torque $T_{o-}$ when current is negative. And Figure 13c shows the comparison of analytical model and experimental result of cogging torque $T_{f}$. It can be found that the analytical model is also consistent with the experimental result.

Differences of between experimental result and analytical model may be caused by the geometrical approximation of magnet poles, specifically spherical crown entity in analytical model whereas block PM poles for the prototype development due to the convenience of fabrication. The stator shell and coil iron cores are geometrically approximated in analytical model for the convenience of computation.

## 7. Conclusions

One novel FSS with spherical actuator is proposed in this paper to reduce the simulator’s redundant inertia force. A hybrid approach is employed to formulate the torque output analytically. Specifically, the Ampere torque is modeled from the equivalent current method, and the cogging torque is obtained from Maxwell stress method. Subsequently, an adaptive PSO method with expectation is proposed for design optimization. It can avoid local minimum value, and obtain global optimization conveniently. The simulation result shows that the optimization can improve the torque generation greatly. The torque comparison between the proposed and the traditional designs shows that the former can greatly improve the output torque and provide torque ripple to perceive the variation of rudder load. Compared with the traditional FSS, the proposed one can reduce the inertia-redundant force and improve the torque servo performance. Based on the optimum values, one research prototype as well as one testbed has been developed. Experiments are conducted on the output torque of the research prototype. The experimental result is compared with the analytical model. It shows that the analytical model fits well with the experimental result. Therefore, the validated analytical torque solution could be used for future studies on motion-control implementations.

## Figures and Tables

**Table 1:** Comparison between different particle nodes number of adaptive PSO algorithm.

No.	Particles Number	Expectation $E_{p}$	Decline Rate $D_{1}$	Decline Rate $D_{2}$
1	5	6.0071	0	0
2	15	0.1053	0	0.9825
3	25	0.1385	0.9825	−0.3152
4	25	1.1356	−0.3152	−7.1996
5	35	0.2068	−7.1996	0.8179

**Table 2:** Key parameters of the prototype.

Stator	Stator radius/(mm)	70
	Number of stator coils poles	12
	Iron core area/(mm${}^{2}$)	425
	Coil area/(mm${}^{2}$)	1000
	Electromagnet area/(mm${}^{2}$)	2050
	Electromagnet angle/(°)	30
	Coil turn number	200
	Coil width/(mm)	20
Rotor	Rotor radius $R_{1}$/(mm)	40
	PM pole parameters $\alpha_{0}$/(°)	45
	PM pole parameters $\alpha_{1}$/(°)	22.5
	Number of rotor PM poles	20
	PM pole thickness/(mm)	15
	PM pole width/(mm)	10
